# Luteolin Shifts Oxaliplatin-Induced Cell Cycle Arrest at G_0_/G_1_ to Apoptosis in HCT116 Human Colorectal Carcinoma Cells

**DOI:** 10.3390/nu11040770

**Published:** 2019-04-02

**Authors:** Chan Ho Jang, Nayoung Moon, Jisun Oh, Jong-Sang Kim

**Affiliations:** School of Food Science and Biotechnology, Kyungpook National University, Daegu 41566, Korea; cksghwkd7@gmail.com (C.H.J.); moonna1008@naver.com (N.M.); j.oh@knu.ac.kr (J.O.)

**Keywords:** flavonoid, luteolin, oxaliplatin, colorectal cancer, anticancer

## Abstract

Certain antioxidative flavonoids are known to activate nuclear factor E2-related factor 2 (Nrf2), a transcription factor that regulates cellular antioxidants and detoxifying response and is reportedly highly activated in many types of cancers. Few studies on the potential undesired effects of flavonoid intake during chemotherapy have been conducted, yet Nrf2 activators could favor cancer cell survival by attenuating chemotherapeutic efficiency. This study aimed to examine if luteolin, an Nrf2 activator, hinders chemotherapeutic activity of oxaliplatin, a potent anticancer agent for colorectal cancer, in HCT116 cells. Luteolin treatment strongly increased the transcriptional activity of the antioxidant response element in HCT116 cells and induced the protein expression of heme oxygenase-1, which were indicative of its Nrf2-inducing potential. Intriguingly, 25 μM luteolin reduced cell viability through apoptotic induction, which was intensified in p53-expressing cells while 1 μM oxaliplatin caused cell cycle arrest at G_0_/G_1_-phase via the p53/p21-dependent mechanism. Moreover, luteolin treatment was found to reduce oxaliplatin-treated p53-null cell viability and colony counts further, thereby demonstrating an additional effect of luteolin in the killing of human colorectal tumor HCT116 cells not expressing functional p53 protein. The findings suggest that luteolin can induce p53-mediated apoptosis regardless of oxaliplatin treatment and may eliminate oxaliplatin-resistant p53-null colorectal cells.

## 1. Introduction

The cellular redox status exists in a dynamic balance between production and elimination of reactive oxygen species (ROS) [1,2,3]. Although a cell undergoing aerobic metabolism is essentially exposed to ROS, the redox balance can be controlled by the cellular antioxidant defense response. However, when ROS production exceeds antioxidant capability, oxidative stress is generated. The intracellular oxidative stress that results from excessive ROS production is associated with cancer development and progression [4,5,6]. The cellular defense against the oxidative stress is mainly achieved by activation of the nuclear factor E2-related factor 2/antioxidant response element (Nrf2/ARE) signaling pathway [7,8,9]. Most chemopreventive compounds including dietary phytochemicals have been reported to remove intracellular ROS by increasing the expression of genes involved in the antioxidant defense system, which, in turn, is activated by Nrf2, a transcription factor liberated from Kelch-like ECH-associated protein 1 (Keap1), translocated to the nucleus, and subsequently bound to the ARE of target genes [10,11,12].

Flavonoids are a group of naturally occurring phenolic compounds that occur in a variety of fruits and vegetables [13,14]. Some flavonoids are well known for exerting chemopreventive effects by upregulating the Nrf2/ARE-mediated cellular defense mechanism against oxidative stress [12,15] and by assisting in the control of cellular redox balance [16,17,18]. In addition, certain dietary flavonoids, including genistein and kaempferol, can induce cell cycle arrest and/or apoptotic cell death of various types of carcinoma cells [19,20,21,22,23,24]. Hence, in different regions of the world, numerous cancer patients consume antioxidative flavonoids, even concurrently with a traditional therapeutic regime, such as chemotherapy and/or radiation therapy, in the anticipation of their synergistic roles of these antioxidants in cooperation with anticancer therapy [25,26].

However, it is conceivable that the flavonoid intake during chemotherapeutic treatment could cause undesired consequences by favoring cancer cell survival and promoting cancer cell growth. This effect is reasonable considering the molecular mechanism that dietary flavonoids activate the Nrf2-mediated cellular defense response against oxidative stress and presumably attenuate the therapeutic efficacy of anticancer drugs in cancer cells. Indeed, the constitutive Nrf2 activation has been found in many types of cancer, and the Nrf2 hyperactivation protected tumor cells from oxidative stress [27]. Furthermore, Nrf2 overexpression enhanced the resistance of cancer cells to chemotherapeutic agents, including cisplatin, doxorubicin, and etoposide [28].

In the present study, we were interested in investigating whether Nrf2-activating flavonoids attenuate or augment the efficacy of chemotherapeutic agents. To narrow down the scope of the work, a human colorectal cancer HCT116 cell line harboring the p53 gene, which is either wild-type or null, was employed. Among the widely-consumed flavonoids tested in this study, luteolin showed the strongest Nrf2-mediated antioxidative activity and was chosen for the elucidation of the molecular mechanism of Nrf2 activation during chemotherapy. Oxaliplatin, a common chemotherapeutic agent for the treatment of colorectal cancer, was concomitantly treated with luteolin in HCT116 cells [29]. The findings would provide insights into understanding the impact of an Nrf2 activator during colorectal cancer chemotherapy.

## 2. Materials and Methods

### 2.1. Chemicals

The flavonoids tested in this study were mostly obtained from Biopurify Phytochemicals Ltd. (Chengdu, Sichuan, China). Epigallocatechin (EGC) and pelargonidin chloride were purchased from Sigma-Aldrich (St. Louis, MO, USA), and delphinidin chloride was obtained from Extrasynthese (Genay, France). Oxaliplatin was purchased from LC Laboratories (Woburn, MA, USA). Dimethyl sulfoxide (DMSO) and all other chemicals, unless otherwise stated, were purchased from Sigma-Aldrich (St. Louis, MO, USA).

### 2.2. Reagents and Antibodies

Dulbecco’s modified Eagle’s medium (DMEM), fetal bovine serum (FBS), penicillin–streptomycin (10,000 U/mL), 4-(2-hydroxyethyl)-1-piperazineethanesulfonic acid (HEPES), minimum essential medium non-essential amino acids (MEM NEAA; 100X), and trypsin–ethylenediaminetetraacetic acid (EDTA)–4Na solution were supplied by Welgene (Gyeongsan, Korea). Cell counting kit-8 (CCK-8; Dojindo Laboratories, Kumamoto, Japan), luciferase assay system (Promega, Madison, WI, USA), polyvinylidene fluoride (PVDF) membranes (Millipore, Burlington, MA, USA), and SuperSignal^®^ West Pico PLUS chemiluminescent substrate kit (Thermo Fisher Scientific, Rockford, IL, USA), cytochrome c (AbFrontier, Seoul, Korea), capase-3 and poly-ADP ribose polymerase (PARP) (both from Cell Signaling Technology, Beverly, MA, USA), and protease inhibitor cocktail (Roche, Beverly, MA, USA) were bought from the suppliers indicated. Antibodies against p53 and p21 were purchased from Santa Cruz Biotechnology (Santa Cruz, CA, USA).

### 2.3. Cell Cultures

HCT116 human colorectal carcinoma cell line carrying wild-type p53 (p53^+/+^) was purchased from the Korean Cell Line Bank (Seoul, Korea). The p53^−/−^ HCT116 cell line was a generous gift from Prof. Young Ho Kim (Kyungpook National University, Daegu, South Korea; originally provided by Dr. Bert Vogelstein, Johns Hopkins University, Baltimore, MD, USA). Both p53^+/+^ and p53^−/−^ HCT116 cell lines were used for the cell viability, colony formation, cell cycle, and Western blot analyses. For the ARE-luciferase reporter gene assay, both types of HCT116 cells were stably transfected with pGL4.37[luc2P/ARE/Hygro] vector (Promega) using TurboFect^TM^ transcription reagents (Thermo Fisher Scientific, Waltham, MA, USA). The transfectants, HCT116-ARE cells, were selected by culturing in the presence of 800 μg/mL of hygromycin B (Sigma-Aldrich).

All the cells were maintained in DMEM supplemented with 10% (*v*/*v*) FBS, 1% (*v*/*v*) penicillin–streptomycin, 2% (*v*/*v*) HEPES, and 1% (*v*/*v*) MEM NEAA in a 5% CO_2_ atmosphere at 37 °C. The medium was changed every 2 or 3 days. Cells were routinely passaged using 0.25% (*w*/*v*) trypsin EDTA when the cells reached about 90% confluency.

### 2.4. Cell Viability Assay

p53^+/+^ and p53^−/−^ HCT116 cells were seeded at a density of 5 × 10^3^ cells per well in a 96-well plate. After a 24-h treatment with oxaliplatin (0–2 μM) or luteolin (5 or 25 μM) in the absence or presence of 1 μM oxaliplatin, the medium was removed, and then 100 μL of phosphate-buffered saline (PBS) and 10 μL of CCK-8 solution were added to each well. A blank control of 10 μL of the CCK-8 solution added to 100 μL PBS alone, was included. The plate was incubated for 1 h, and absorbance was measured at 450 nm using a Sunrise^TM^ microplate reader (Tecan Group Ltd., Männedorf, Switzerland).

### 2.5. Antioxidant Response Element (ARE)-Luciferase Reporter Gene Assay

p53^+/+^ and p53^−/−^ HCT116-ARE cells were seeded at a density of 5 × 10^5^ cells per well in a 6-well plate. After a 24-h treatment with luteolin and/or oxaliplatin, cells were harvested in an ice-cold PBS solution and lysed with reporter lysis buffer. Lysates were mixed with luciferase assay reagent (Promega), at room temperature. Each mixture was transferred to individual wells of a 96-well plate and immediately measured using a Glomax^TM^ 96 microplate luminometer (Promega). Protein was quantified by the Bradford assay, and the resultant values were used to normalize the luciferase data.

### 2.6. Colony Formation Assay

p53^+/+^ and p53^−/−^ HCT116 cells were seeded at a density of 1 × 10^2^ cells per well in a 6-well plate coated with Matrigel, for the colony formation assay. The cells were treated with 5 or 25 μM luteolin in the absence or presence of 1 μM oxaliplatin for 6 days. Cell colonies formed in each well were optically visualized by crystal violet staining. The cell colonies were imaged using an HP Scanjet 5550c (Hewlett-Packard, Palo Alto, CA, USA) and counted using ImageJ software version 1.50 for Windows (National Institutes of Health, Bethesda, MD, USA).

### 2.7. Cell Cycle Analysis

p53^+/+^ and p53^−/−^ HCT116 cells were seeded at a density of 2 × 10^6^ cells in a 100-mm dish. After a 24-h treatment with luteolin and/or oxaliplatin, cells were harvested, washed twice with PBS, and fixed with 70% cold ethanol at −20 °C overnight. The cells were washed twice with PBS, suspended in PBS, and transferred to fluorescence-activated cell sorting (FACS) tubes. Then, 400 μL of propidium iodide (50 μg/mL) was added to the tubes, and the cells were incubated at room temperature for 30 min. The cells were analyzed for DNA content by flow cytometry using the FACSAria III^TM^ cell sorter (BD Biosciences, Franklin, NJ, USA). Staurosporine (2 μM) was used as a positive control to induce apoptosis.

### 2.8. Western Blot Analysis

p53^+/+^ and p53^−/−^ HCT116 cells were seeded at a density of 2 × 10^6^ cells in a 100-mm dish. After a 24-h treatment with luteolin and/or oxaliplatin, cells were harvested in a cold PBS solution, lysed with radioimmunoprecipitation assay (RIPA) buffer, and the protein samples were isolated from the lysates. Identical protein amounts were subjected to 10% sodium dodecyl sulfate-polyacrylamide gel electrophoresis and then transferred to PVDF membranes. The membranes were blocked with 1% bovine serum albumin and sequentially incubated with primary and secondary antibodies. The antibody-bound proteins were visualized using the SuperSignal^®^ West Pico PLUS chemiluminescent substrate kit (Thermo Fisher Scientific) and ImageQuant^TM^ LAS 4000 Mini (GE Healthcare Life Sciences, Little Chalfont, UK). Intensities of protein bands were determined by Image Studio Lite version 5.2 (LI-COR Biotechnology, Lincoln, NE, USA).

### 2.9. Statistical Analysis

The data were statistically analyzed using SPSS version 23.0 for Windows (SPSS, Inc., Chicago, IL, USA), and are presented as mean ± standard deviation (SD) of three independent experiments (*n* = 3). Comparisons were conducted using one-way analysis of variance (ANOVA) followed by a post-hoc Tukey’s honestly significant difference (HSD) test or Duncan’s multiple-range test. The significant difference compared with the control was indicated by an asterisk or different alphabetical letters at *p* < 0.05.

## 3. Results

### 3.1. Cytotoxicity of Oxaliplatin

Oxaliplatin is well known for treating colorectal cancer by preventing DNA replication and transcription, causing cell death [29]. To test the cytotoxicity of oxaliplatin, the cell viabilities of p53^+/+^ and p53^−/−^ HCT116 cells treated with oxaliplatin were analyzed by a CCK-8 assay (Supplementary Figure S1). Oxaliplatin at concentrations of ≥0.5 μM reduced the viability of p53^+/+^ HCT116 cells to approximately 90%, whereas p53^−/−^ HCT116 cells needed ≥2 μM oxaliplatin to achieve the same effect, indicating that p53^+/+^ HCT116 cells were more susceptible to oxaliplatin than p53^−/−^ HCT116 cells. Hence, 1 μM oxaliplatin was selected for subsequent studies of flavonoid intake during oxaliplatin-based chemotherapy.

### 3.2. ARE-Luciferase Activity of Flavonoids

**Figure 1:**
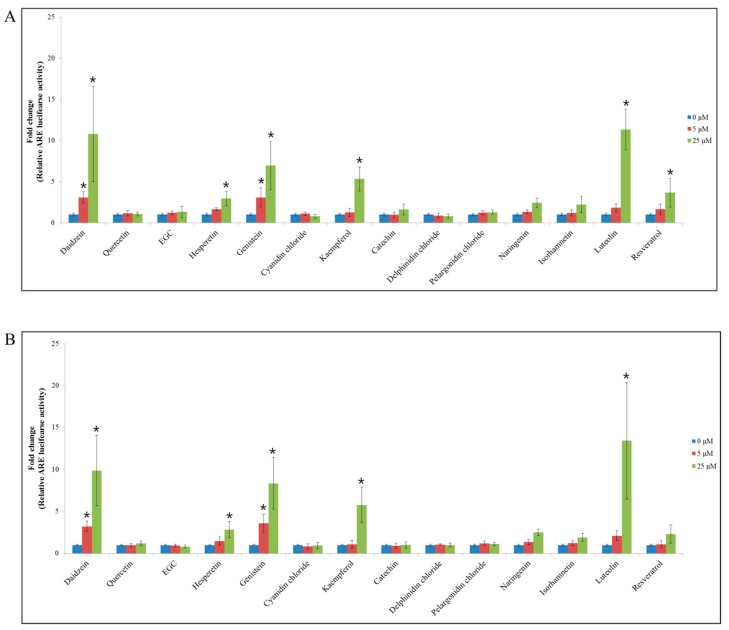
ARE-luciferase activity for 14 flavonoids in p53^+/+^ and p53^−/−^ HCT116 cells. ARE-luciferase activity of 14 flavonoids at 5 or 25 μM in p53^+/+^ HCT116 cells (**A**) and p53^−/−^ HCT116 cells (**B**) after 24-h treatment. The ARE-luciferase activity was normalized to the total protein content. Data are expressed as mean ± SD of three independent experiments (*, a significant difference compared to the control group at *p* < 0.05).

All 14 flavonoids were individually tested for their ability to activate the Nrf2/ARE signaling pathway, by conducting the ARE-luciferase reporter gene assay in p53^+/+^ and p53^−/−^ HCT116–ARE cells (Figure 1). Among the flavonoids tested, at 25 μM, daidzein, genistein, kaempferol, and luteolin significantly induced the ARE–luciferase reporter in both p53^+/+^ and p53^−/−^ HCT116–ARE cells compared with the control. As a result the ARE-luciferase activity was increased by 10.8- (daidzein), 7.0- (genistein), 5.3- (kaempferol), and 11.3-fold (luteolin) in p53^+/+^ HCT116–ARE cells (Figure 1A), and by a corresponding 9.9-, 8.4-, 5.8-, and 13.4-fold in p53^−/−^ HCT116–ARE cells (Figure 1B). These data showed that luteolin preferentially stimulated the Nrf2/ARE signaling pathway in both p53^+/+^ and p53^−/−^ HCT116–ARE cells than the other flavonoids.

**Figure 2:**
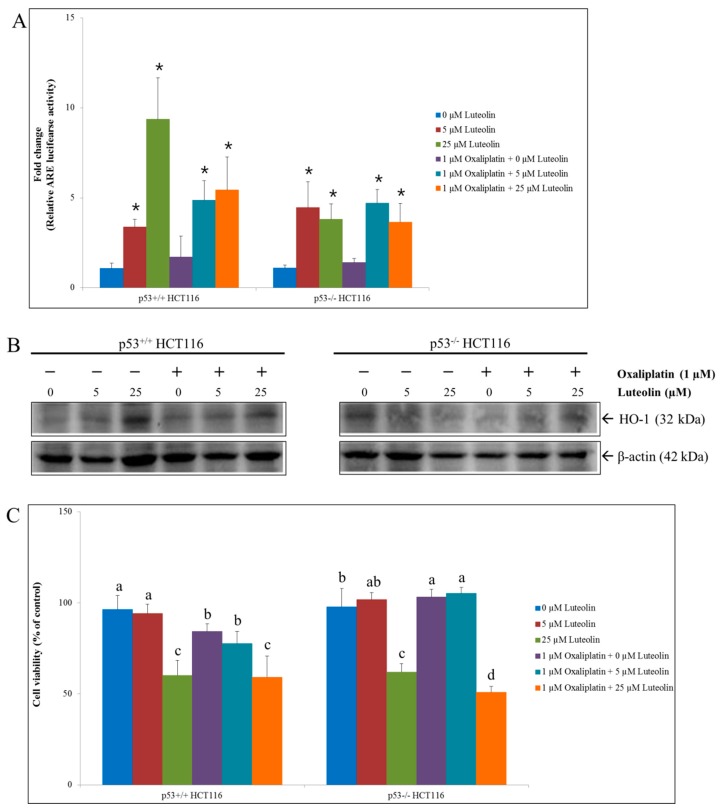
ARE-luciferase activity, HO-1 protein expression, and cell viability in p53^+/+^ and p53^−/−^ HCT116 cells in the presence of luteolin and/or oxaliplatin. Cells were seeded in a 6-well plate, a 100-mm dish, or a 96-well plate and treated with 5 or 25 μM luteolin in the absence or presence of 1 μM oxaliplatin at for 24 h, followed by measurement of the ARE activity, Western blot analysis for HO-1 expressions or cell viability assay. (**A**) Relative ARE-luciferase activity. Data are expressed as mean ± SD of three independent experiments. A significant difference compared to the control group at *p* < 0.05 was indicated by an asterisk. (**B**) Representative Western blots of protein expression changes of HO-1. (**C**) Relative cell viability assayed using a CCK-8 kit. Data are expressed as mean ± SD of three independent experiments. A significant difference compared among the groups at *p* < 0.05 was indicated by different alphabetical letters.

The potential of luteolin to activate the Nrf2-mediated antioxidant response in HCT116 cells was further examined by determining the expression of heme oxygenase-1 (HO-1), an archetypical inducible antioxidant enzyme that is regulated by the Nrf2/ARE signaling pathway. Luteolin increased the expression of HO-1 in p53^+/+^ HCT116 cells only while it induced ARE activity in both p53^+/+^ and p53^−/−^ HCT116 cells (Figure 2A,B), consistent with the findings reported by other studies [10,15,16,17,18,30]. On the contrary, HO-1 expressions were not affected by oxaliplatin treatment in both cell lines (Figure 2B).

### 3.3. HCT116 Cell Viability by Luteolin and/or Oxaliplatin Treatment

To examine if Nrf2-activating luteolin compromises the efficacy of oxaliplatin in colorectal cancer cells, the HCT116 cell viability was determined following individual or combinatorial treatment with luteolin at 5 or 25 μM and oxaliplatin at 1 μM for 24 h (Figure 2C).

The individual treatment with luteolin at 25 μM remarkably decreased the viability of both p53^+/+^ and p53^−/−^ HCT116 cells, indicating that luteolin effectively inhibited the cell growth. In addition, oxaliplatin alone and the combination of luteolin and oxaliplatin significantly decreased the p53^+/+^ HCT116 cell viability. However, the survival rate of p53^−/−^ HCT116 cell was not affected by oxaliplatin treatment. The cell viability reduced by luteolin was further decreased by co-treatment with oxaliplatin in the absence of p53 expression. These observations demonstrate that the anticancer efficacy of oxaliplatin was dependent upon the existence of p53 while a cell growth inhibitory effect of luteolin was prominent in both cell types.

### 3.4. Mechanism of Cell Growth Inhibition by Luteolin and Oxaliplatin

**Figure 3:**
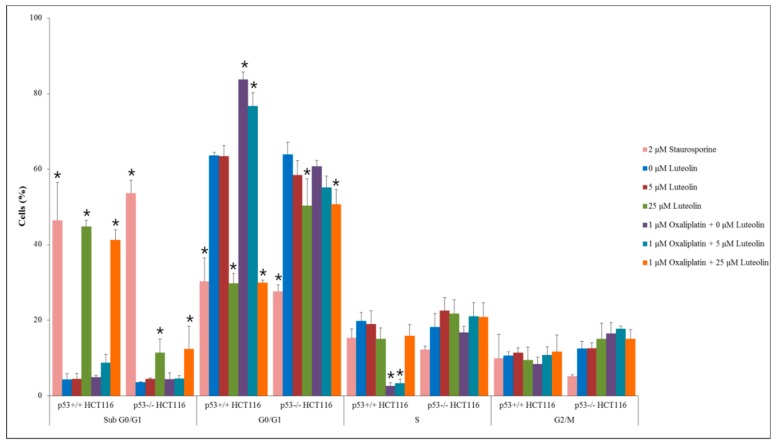
Cell cycle analysis in p53^+/+^ and p53^−/−^ HCT116 cells. Cells were seeded in a 100-mm dish and treated with 5 or 25 μM of luteolin in the absence or presence of 1 μM oxaliplatin for 24 h. Staurosporine (2 μM) was used as a positive control to induce apoptosis. Data are expressed as mean ± SD of three independent experiments (*, a significant difference compared to the control group at *p* < 0.05).

To investigate how luteolin and/or oxaliplatin inhibited cancer cell growth, flow cytometric analysis was performed on p53^+/+^ and p53^−/−^ HCT116 cells (Figure 3). Treatment of HCT116 cells with luteolin at 25 μM led to a significant increased proportion of cells at the sub-G_0_/G_1_-phase, an indicator of apoptosis, compared with the control, regardless of oxaliplatin treatment, even though the proportion of cells in sub-G_0_/G_1_-phase was prominently higher in the p53^+/+^ HCT116 cell population than p53^−/−^ HCT116 cell population. It suggests that luteolin induced apoptosis, which was dominantly dependent on functional p53. In contrast, oxaliplatin significantly increased the cell population arrested at the G_0_/G_1_-phase and decreased the population at the S-phase relative to the control, in p53^+/+^ HCT116 cells but not in p53^−/−^ HCT116 cells, suggesting that oxaliplatin induced cell cycle arrest in a p53-dependent manner.

**Figure 4:**
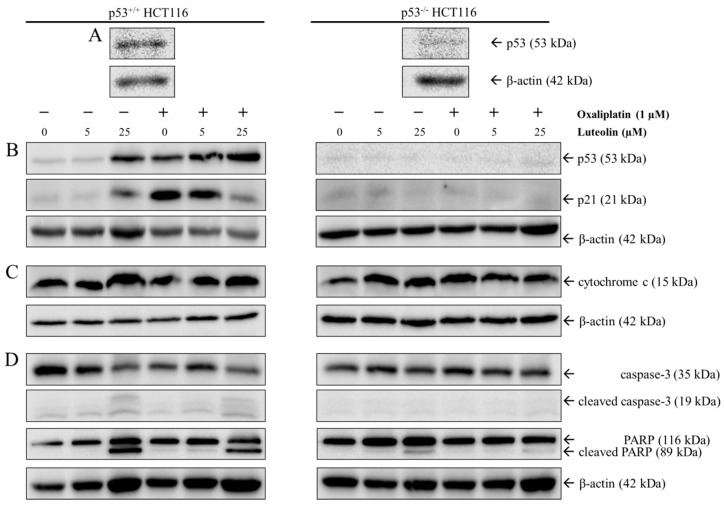
Western blot analysis in p53^+/+^ and p53^−/−^ HCT116 cells. Representative Western blots of p53 in p53^+/+^ and p53^−/−^ HCT116 cells (**A**). Cells were seeded in a 100-mm dish and treated with 5 or 25 μM of luteolin in the absence or presence of 1 μM oxaliplatin for 24 h. Representative Western blots of protein expression changes of p53 and p21 (**B**), cytochrome c (**C**), and cleaved caspase-3 and poly-ADP ribose polymerase (PARP) (**D**) in p53^+/+^ and p53^−/−^ HCT116 cells.

That both luteolin-induced apoptosis and oxaliplatin-induced cell cycle arrest is p53-dependent, were further verified by Western blot analysis using p53^+/+^ and p53^−/−^ HCT116 cells following 24-h treatment with luteolin and/or oxaliplatin (Figure 4). As expected, p53 protein was abundantly expressed in p53^+/+^ HCT116 cells but was barely detected in p53^−/−^ HCT116 cells (Figure 4A). Within p53^+/+^ HCT116 cells, the expressions of p53 and its downstream *gene p21*, a cyclin-dependent kinase (CDK) inhibitor that promotes cell cycle arrest, were considerably increased by oxaliplatin and 25 μM luteolin (Figure 4B). In addition, luteolin increased the expressions of apoptosis-associated proteins, such as cytochrome c, cleaved caspase-3, and cleaved PARP in p53^+/+^ HCT116 cells, regardless of oxaliplatin treatment (Figure 4C,D). Interestingly, the combinatorial treatment of oxaliplatin and luteolin reduced p21 protein expression in p53^+/+^ HCT116 cells, but not in p53^−/−^ HCT116 cells, compared to the treatment with oxaliplatin alone (Figure 4B). These observations were supportive of the result mentioned above that the combinatorial treatment of oxaliplatin and luteolin further decreased p53^−/−^ HCT116 cell viability, which may be attributable to the partial inhibition of oxaliplatin-induced cell cycle arrest by luteolin in a p53-dependent manner while luteolin and oxaliplatin induced apoptosis and cell cycle arrest, respectively.

### 3.5. Colony-Forming Ability of HCT116 Cells Treated with Luteolin and/or Oxaliplatin

**Figure 5:**
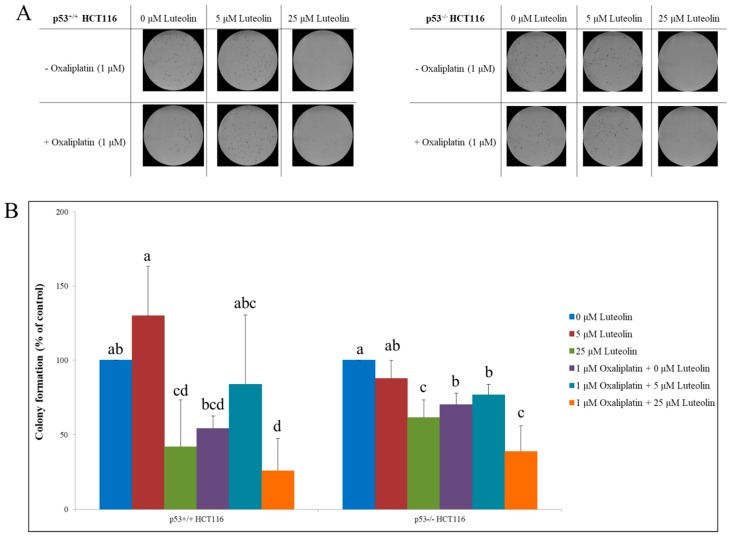
Colony-forming ability of p53^+/+^ and p53^−/−^ HCT116 cells in the presence of luteolin and/or oxaliplatin. Cells were seeded in a 6-well plate and treated with luteolin with or without oxaliplatin for 6 days. (**A**) Colonies were stained with crystal violet, imaged and counted. Representative images of colonies formed in p53^+/+^ HCT116 (left) and p53^−/−^ HCT116 cultures (right). (**B**) Relative colony formation ability in HCT116 cells was quantified. Data are expressed as mean ± SD of three independent experiments. A significant difference compared among the groups at *p* < 0.05 was indicated by different alphabetical letters.

The effects of luteolin, oxaliplatin, and their combination on the proliferative properties of both p53^+/+^ and p53^−/−^ HCT116 cells were examined using a colony formation assay [31]. The cells grown in discrete colonies were treated with luteolin (5 or 25 μM) in the absence or presence of oxaliplatin (Figure 5B,C). In comparison to the control, the individual treatment with either luteolin at 25 μM or oxaliplatin significantly inhibited the colony growth in both types of cells; however, the combinatorial treatment did not cause further augmentation of colony growth inhibition by each treatment in p53^+/+^ HCT116 cells. Interestingly, the growth of colonies formed from p53^−/−^ HCT116 cells was significantly suppressed by the combinatorial treatment of luteolin and oxaliplatin. In summary, oxaliplatin and the high dose of luteolin decreased cancer cell growth individually, but the combinatorial treatments did not synergistically reduce cancer cell proliferation in the presence of functional p53.

## 4. Discussion

Based on increasing epidemiological and laboratory evidence, regular intake of flavonoid-rich food is most likely to reduce the risk of colorectal cancer by interfering with different cancer development stages, such as the protection of DNA from oxidative stress, the inhibition of carcinogen activation, and the activation of carcinogen-detoxifying systems [32,33,34,35,36,37].

However, it has been recently reported that certain dietary flavonoids could promote cancer cell proliferation via activation of the Nrf2/ARE signaling pathway [38,39,40], which is known to be involved in resistance of cancer cells or cancer stem cells to chemotherapy or radiotherapy [27,41,42,43,44]. Therefore, it is reasonably predictable that the consumption of Nrf2-activating flavonoids during chemotherapeutic treatment could result in a diminished chemotherapeutic efficacy [29,45].

In the present study, we examined the in vitro effect of luteolin, an Nrf2-activating flavonoid, on HCT116 colorectal cancer cell growth in combination with oxaliplatin treatment. The findings from this study demonstrated that (1) luteolin activated the Nrf2/ARE/HO-1 signaling pathway and promoted p53-dependent and independent apoptotic pathways regardless of the absence or presence of oxaliplatin; (2) oxaliplatin caused cell cycle arrest at the G_0_/G_1_-phase in an exclusively p53-dependent manner; (3) luteolin-induced HO-1 upregulation hindered oxaliplatin-induced cell cycle arrest under the existence of p53 protein, and (4) luteolin-induced cell growth inhibition was augmented by oxaliplatin in the absence of functional p53 protein.

Oxaliplatin is one of the chemotherapeutic drugs used for treating several types of cancers, including colorectal cancer, by inducing cell cycle arrest at checkpoints such as the G_0_/G_1_-phase, the G_2_/M-phase, and metaphase checkpoints via the prevention of DNA replication and transcription that causes cell death [29,46]. Our results exhibited that oxaliplatin had a differential effect on HCT116 cells, depending on the existence of functional p53; that is, functional p53-expressing cells were more vulnerable to oxaliplatin treatment. Considering that oxaliplatin treatment highly increased cell population arrested at the G_0_/G_1_-phase and p21 protein expression only in HCT116 cells, the cell cycle modulating the activity of oxaliplatin in HCT116 cells is likely mediated through the p53–p21 axis.

Conversely, 25 μM luteolin significantly activated the Nrf2/ARE/HO-1 signaling pathway in both types of HCT116 cells, which was consistent with the previous studies, confirming the strong antioxidative activity of luteolin [47,48]. Moreover, luteolin treatment considerably increased the cell population at the sub-G_0_/G_1_-phase, particularly in p53-expressing HCT116 cells compared with p53-null HCT116 cells, suggesting that at the concentration used in this study, luteolin caused HCT116 cell death through p53-mediated apoptotic pathways.

Since both oxaliplatin-induced cell cycle arrest and luteolin-induced apoptotic cell death of HCT116 cells seemed p53-dependent, the functional interaction between the two agents was presumed. In this study, we found that the reduction of cell viability which was attributable to apoptotic cell death by the high dose of luteolin was not further influenced by oxaliplatin treatment in the existence of p53. These findings imply that there was no synergistic effect from the combinatorial treatment of oxaliplatin and luteolin in killing HCT116 colorectal cancer cells. In other words, oxaliplatin-induced cell cycle arrest and luteolin-induced apoptosis might involve separate pathways in HCT116 cells. Alternatively, the apoptotic activity of luteolin may dominate the cell cycle, controlling oxaliplatin activity in wild-type, p53-expressing cells.

Moreover, luteolin treatment increased HO-1 expression in p53-expressing HCT116 cells regardless of oxaliplatin treatment, via Nrf2/ARE activation and, subsequently, inhibited oxaliplatin-induced cell cycle arrest, with reduction of p21 expression. It suggests that luteolin may decrease the cell cycle arrest by oxaliplatin, via the activation of the Nrf2/ARE/HO-1 signaling pathway in p53-expressing colorectal cancer cells. There is accumulating evidence that the induction of HO-1 functions negatively by promoting tumor resistance through the reduction of p21 protein expression in colon cancer cells [49]. As multiple studies have demonstrated, it is widely accepted that Nrf2 and p53 are negatively correlated in cell survival and death [50,51,52]. However, our study implied that p53 could play an important role in Nrf2/ARE/HO-1 activation and apoptotic cell death. Luteolin-induced apoptosis is presumed to be magnified in association with suppression of p21 protein expression, which, at least in part, is achieved by HO-1 upregulation. However, the precise mechanism of an interrelationship between the pathways awaits further study.

One of the interesting observations in this study was that luteolin treatment tended to release oxaliplatin-induced cell cycle arrest in the presence of p53 protein, which is consistent with the apparent reduction of p21 protein expression in p53-expressing HCT116 cells treated with the combination of oxaliplatin and the high dose of luteolin. There is mounting evidence indicating that the manipulation of the cell cycle may prevent or induce an apoptotic response, depending on the cellular context [53,54]. In terms of a p21-dependent signaling pathway, the expression of p53 protein is required for the p21 protein expression, which, in turn, can induce the cell cycle arrest by interacting with the cyclin D/CDK4 complex in human cancer [53,55,56,57]. Based on the findings from this study, it is thought that luteolin may suppress the p53/p21-mediated pathway by which oxaliplatin induces cell cycle arrest in colorectal cancer cells, thereby switch cell cycle arrest by oxaliplatin into apoptosis.

The p53-null HCT116 cells were found to be less sensitive to oxaliplatin compared with the cells expressing normal p53 proteins. However, luteolin-treated p53-null HCT116 cells appeared to become more vulnerable to oxaliplatin in comparison to the cells not treated with luteolin. This observation is suggestive of the critical function of p53 in apoptosis and cell cycle arrest, in response to luteolin and/or oxaliplatin. Although the molecular participants and their networking related to the functional p53 protein as regards the effectiveness of luteolin, on oxaliplatin-induced chemotherapeutic efficacy remains to be further investigated. Dietary luteolin intake during oxaliplatin-based chemotherapy would at least not be deleterious to the anticancer efficacy of oxaliplatin but, rather, could be additively working in colorectal cancer, carrying malfunctioning p53 protein by augmenting cancer cell growth inhibition.

The p53 mutations encoding abnormal or malfunctioning p53 proteins frequently occur in many types of cancers, including colon cancer [58,59,60], and could contribute to cancer progression and development by losing wild-type p53 tumor suppressor activity [59,61]. Hence, studying the role of antioxidative luteolin against or for chemotherapeutic effectiveness of oxaliplatin in accordance with various types of p53 mutations in cancer cells would be worthwhile for practical applications.

## 5. Conclusions

**Figure 6:**
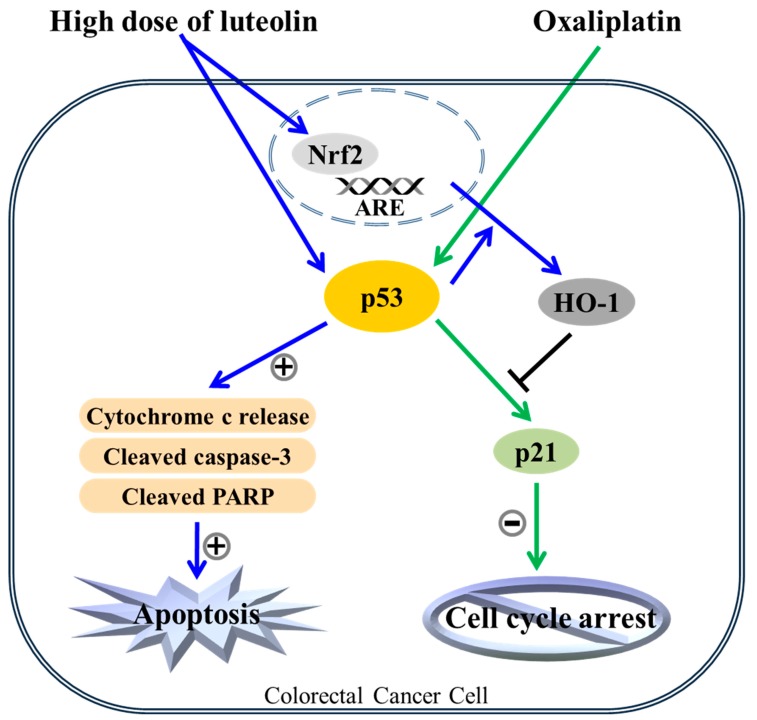
The apoptotic mechanism of the combinatorial treatment of luteolin and oxaliplatin. Both luteolin-induced apoptosis and oxaliplatin-induced cell cycle arrest in HCT116 human colorectal cancer cells were dependent on p53 function. Luteolin-induced apoptosis might override the oxaliplatin-induced cell cycle arrest, possibly through the increased HO-1 expression in p53-expressing cells. In addition, luteolin-induced p53-null HCT116 cell death could be intensified by oxaliplatin treatment through an unknown mechanism(s).

In conclusion, we showed for the first time that luteolin, a naturally occurring Nrf2 activator, can enhance the chemotherapeutic effect of oxaliplatin in HCT116 human colorectal cancer cells, potentially by shifting oxaliplatin-induced cell cycle arrest to apoptosis. The present study revealed that luteolin treatment induced Nrf2/ARE/HO-1 activation in HCT116 human colorectal cancer cells. In addition, luteolin and oxaliplatin caused apoptosis and cell cycle arrest, respectively, in a p53-dependent manner, with no synergistic effect. Moreover, luteolin-induced Nrf2/ARE/HO-1 activation negatively regulated oxaliplatin-promoted p53 signal transduction, and oxaliplatin-induced cell cycle arrest was seemingly disturbed by luteolin-induced HO-1 upregulation. Overall, these results suggest that luteolin could strengthen the anticancer activity of oxaliplatin, one of the chemotherapeutic agents, in human colorectal carcinoma cells in a p53-dependent manner. Thus, dietary consumption of flavonoids with antioxidant enzyme-inducing activity may be recommended for colorectal cancer patients carrying wild-type p53 (Figure 6). Furthermore, these findings provide novel insight into the further understanding of the mechanisms underlying the enhanced chemotherapeutic effect of oxaliplatin and luteolin in colorectal cancer. However, further studies, including the in vivo impact of luteolin on oxaliplatin-based chemotherapy are required to validate the current findings.

## Supplementary Materials

The following are available online at https://www.mdpi.com/2072-6643/11/4/770/s1, Figure S1: Cytotoxicity of oxaliplatin against p53^+/+^ and p53^−/−^ HCT116 cells. Cells were seeded at a density of 5 × 10^3^ cells per well in a 96-well plate and treated with various concentrations of oxaliplatin for 24 h. Cytotoxicity was determined by a CCK-8 assay. Data are expressed as mean ± SD of three independent experiments (*, a significant difference compared to the control group at *p* < 0.05).
