# Supplementary material for: Luteolin Shifts Oxaliplatin-Induced Cell Cycle Arrest at G0/G1 to Apoptosis in HCT116 Human Colorectal Carcinoma Cells
Source: Nutrients. 2019 Apr 2;11(4):770. doi: 10.3390/nu11040770 (PMC6521147; doi:10.3390/nu11040770)
Supplement: Supplementary file 1 [file nutrients-11-00770-s001.pdf]

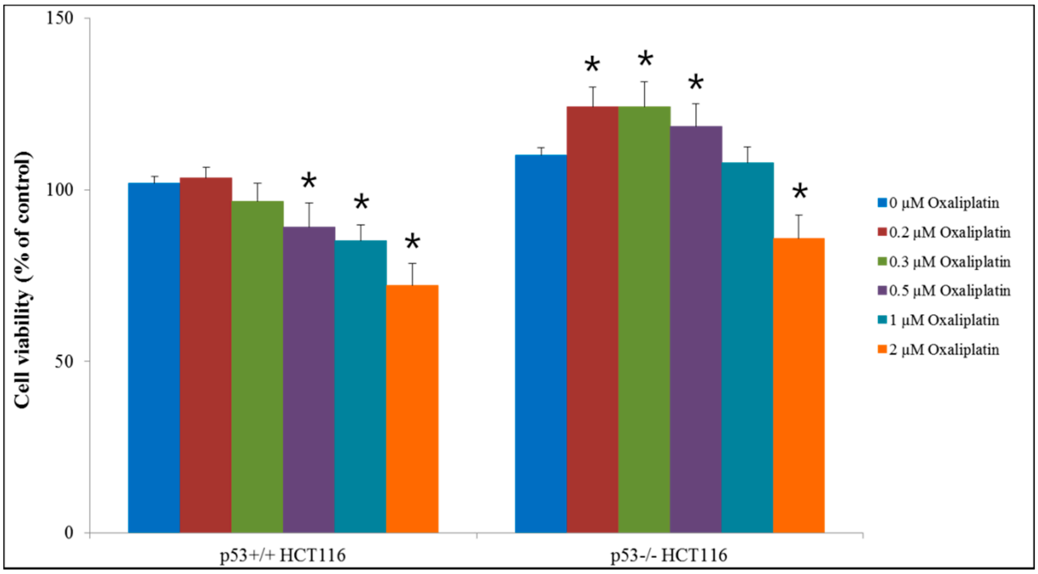

Supplementary Figure S1. Cytotoxicity of oxaliplatin against $p53^{+/+}$ and $p53^{-/-}$ HCT116 cells. Cells were seeded in a 96-well plate and treated with various concentrations of oxaliplatin for 24 h. Cytotoxicity was determined by a CCK-8 assay. Data are expressed as means ± SD ($N = 3$). A significant difference is indicated by an asterisk, * at $p < 0.05$ compared with the control group.
